# Screening Tools as a Predictor of Injury in Gymnastics: Systematic Literature Review

**DOI:** 10.1186/s40798-021-00361-3

**Published:** 2021-10-11

**Authors:** Ross Armstrong, Nicola Relph

**Affiliations:** 1 Rehabilitation and Healthy Lives Research Group, Institute of Health, University of Cumbria, Carlisle, Cumbria CA1 2HH England; 2 Promoting Population Musculoskeletal Health Research Group, Faculty of Health and Social Care, Edge Hill University, Ormskirk, Lancashire L39 4QP England

**Keywords:** Gymnastics, Height, Weight, Body mass index, Injury, Pain

## Abstract

**Background:**

Gymnastics requires a high level of physical ability and technical skill which utilises short sets of athleticism and artistry to perform complex and intense movements which can overload musculoskeletal tissues and result in acute injuries which can develop into chronic injuries. The aim of this systematic literature review was to investigate which screening tools predict injury in gymnasts and encompasses all genres, levels and ages.

**Methods:**

An electronic search of seven databases from their inception until March 2021 was conducted. The databases were the Allied and Complementary Medicine Database, CINAHL, eBook Collection (EBSCOhost), MEDLINE, Cochrane Database of Systematic Reviews, SPORTDiscus and PEDro (the Physiotherapy Evidence Base). A combination of the following search terms was used: (1) Gymnastics AND injury AND Screening, (2) Screening AND Gymnastics and (3) Musculoskeletal AND Screening AND Gymnastics. These terms were searched in all text, abstract, title and subject terms. Studies were assessed using a 20-point scoring tool.

**Results:**

The mean methodological quality score was 13.1 points (range 10–17 points). Range of motion, anthropometric and postural measurements, hypermobility, clinical diagnostic tests, movement screening tools, muscle strength, power and endurance were reported in the included studies. Some evidence existed for screening measurement of height and mass as taller and heavier gymnasts might be more susceptible to injury; however, the different methodologies utilised and lack of acknowledgment of confounding variables limit the clinical relevance of these findings.

**Conclusions:**

Height and mass should be recorded during the screening process. A lack of heterogeneity in study methodology prevented a meta-analysis. Studies were limited by a lack of prospective injury design, poor injury definition, self-reporting of injury and only 2 studies reported reliability of screening tools. Further research is required to determine the role of injury screening in gymnastics.

*Registration*: The review protocol was registered with the International Prospective Register of Systematic Reviews (PROSPERO) with the registration number CRD42020218339.

## Key Points

- Taller and heavier gymnasts might be more susceptible to injury.
- Studies have investigated a range of screening measurements.
- There is a need for prospective studies that define injury and measure the reliability of screening tools used.

## Background

Gymnastics requires a high level of physical ability and technical skill [1] which utilises short sets of athleticism and artistry to perform complex and intense movements. These athletic characteristics include strength, speed, power, agility, cardiovascular endurance, flexibility, coordination and balance. Gymnastics can involve several disciplines including rhythmic, artistic, trampoline, acrobatic and aerobic gymnastics all of which have different physical demands. Gymnastics requires repetitive movements which can overload joints and result in acute injuries which can develop into chronic injuries. These loads combined with movements that regularly exceed normal anatomical range can potentially result in injury. In aerobic and acrobatic gymnastics, the high volume of throws and catches in the jump elements creates high impact loading in lower extremity joints [2]. As the demands and rewards of competition increase it is likely that gymnasts will continue to work on the periphery of a sustainable training load and without sufficient rest the injury risk will remain high.

Injury rates of 1.08/1000 h participation have been reported in rhythmic gymnastics [3]. Injury rates per 1000 h for artistic gymnastics participation range from 0.427 [4] in lower-level female gymnasts to 22.7 [5] in intercollegiate competition gymnasts. Within artistic gymnastics the floor apparatus is associated with the greatest injury risk [6–8]. Gymnastic movements rely heavily on integration via the kinetic chain with skills such as handsprings and walkovers involving spine and hip extension and shoulder hyperflexion [9]. Injury surveillance of 3 Olympic games has identified that the lower limb (62.8%), trunk (23.1%) and upper limb (14.1%) as the regions most susceptible to injury with the ankle the most prominent location (21.8%) [10]. The dominance of lower limb injury is supported across competitive  levels in further studies [6, 7, 11]. Therefore, there is a high risk of injury in gymnastics regardless of discipline and level. Injury can have a detrimental impact on a gymnast’s health and well-being, and therefore, injury prevention interventions including screening are essential.

The Van Mechelen model of injury prevention [12] and the development of injury prevention programmes requires injury surveillance to identify best practice and potential interventions. One such intervention is the use of screening tools to identify athletes that are at risk of injury [13–16]. The determination and implementation of effective screening tools may have a positive physical and psychological impact on gymnasts by potentially reducing injury risk via the implementation of injury prevention programmes. The current systematic literature review is the first to investigate which screening tools can predict injury in gymnasts and encompasses all genres, levels and ages. A meta-analysis was also proposed to synthesise similar data sets where appropriate.

## Methods

The review protocol was registered with the International Prospective Register of Systematic Reviews (PROSPERO) [17] with the registration number CRD42020218339.

### Search Strategy

A systematic literature search was conducted to obtain articles regarding screening tools that can potentially predict injury in gymnasts from the inception of seven databases until March 2021. The databases were the Allied and Complementary Medicine Database (AMED), CINAHL, eBook Collection (EBSCOhost), MEDLINE, Cochrane Database of Systematic Reviews, SPORTDiscus and PEDro (the Physiotherapy Evidence Base). A combination of the following search terms was used: (1) Gymnastics AND injury AND Screening, (2) Screening AND Gymnastics and (3) Musculoskeletal AND Screening AND Gymnastics. These terms were searched in all text, abstract, title and subject terms. Reference lists of acquired articles were screened to find additional articles, and duplicates were removed.

### Study Selection

The titles and abstracts of the search returned articles were reviewed by the first author (RA) to identify potential relevance using a two-stage process. The first stage involved the classification of articles as relevant, potentially relevant or irrelevant. During this stage, irrelevant articles were excluded, and articles that met the inclusion criteria were retained for further analysis. The second stage involved the review of the full text of relevant and potentially relevant articles by two reviewers (RA and NR). Both reviewers formulated comments regarding the suitability of articles using the checklist of five inclusion criteria and then met to determine final inclusion via reviewing these comments. Any potential disagreements regarding the inclusion were referred to a third reviewer to determine final inclusion. Studies were included if they were (1) full text, (2) in the English language, (3) used a screening tool and/or physical measurement, (4) the population was gymnasts and (5) injury or pain occurrence was reported either retrospectively or prospectively. This review only included screening tools that can be utilised  in the field. Therefore, studies that utilised equipment such as thermal imaging, force plates and computerised dynamic posturography were excluded as they were deemed to be laboratory-based and limited in the practical application of gymnastic injury screening.

### Data Extraction

Two reviewers (RA and NR) independently extracted data from each article. The following information was extracted if available: study design (prospective or retrospective), level of evidence, location of testing, inclusion and exclusion criteria, subject characteristics (age, sex, height, weight); screening tool and/or physical measurements recorded; reliability and validity of screening tool and/or physical measurements and method of injury collection including retrospective/prospective injury collection, definition of injury, individual diagnosing injury, statistical analysis of injury measure, percentage of missing data or withdrawals, outcome measures and identification of confounders.

### Methodological Quality

A previous review of injury screening tools in dance [18] utilised a 20-point scoring system and the authors provided permission for use of this tool in the current review. This scoring tool was developed from a previously published screening tool in team sports [19] and modified version Cochrane Group on Screening and Diagnostic Test Methodology (Cochrane methods) [20]. The scoring system is outlined in Table 1. For study design, those studies that included both retrospective and prospective injury data collection were awarded 1 point. The level of evidence devised from the Oxford Centre for Evidence-Based Medicine ranged from 1 to 5, with 1 the lowest and 5 the highest score. Both inclusion and exclusion criteria had to be stated to score 1 point and setting information needed to include the name of the venue for 1pt. Demographic information needed to contain a minimum of age and gender to score 1pt. The screening tool needed to be described sufficiently to allow replication to score 1pt. An effective injury definition and diagnosis by an appropriate professional (e.g. physiotherapist and/or doctor) is essential [18]; therefore, the presence of both criteria was awarded 1pt each. The methodological score based on statistical analysis was divided into two separate questions. The study was awarded 1 point if it had included an inferential statistical analysis of any kind. However, the study was awarded an additional point if a regression model or risk measurement had been applied; in the current review, this included linear regression models, logistical regression models, Cox regression models, odds ratio (OR) analysis and relative risk (RR) analysis. This aspect of the methodological quality score allowed differentiation between studies that consider the injury screening tool predictive capability and those that did not. The studies which considered only the ability of the screening tool to identify the differences between the injured and non-injured groups were not awarded this additional point. The reliability of tools is a fundamental component of an effective methodology, and therefore, 1 point was awarded for studies that reported reliability from previous studies and 2 points for those that reported the reliability from the actual study data. Those studies that reported the withdrawal of participants and provided information regarding missing data were awarded 1pt. For outcome measures studies were awarded 1 point if the outcome measures were clearly reported and studies were awarded 1 point if the confounders were reported.

**Table 1:** Methodological quality score for each study

Study	Design^a^ [1]	Level of evidence^b^ [5]	Selection criteria^c^ [1]	Setting^d^ [1]	Demographic information^e^ [1]	Description of screening tool^f^ [2]	Injury definition^g^ [1]	Injury diagnosis^h^ [1]	Statistical analysis^i^ [1]	Predictive statistical analysis^j^ [1]	Reliability of index test^k^ [2]	Percentage missing^l^ [1]	Outcome^m^ [1]	Confounders^n^ [1]	Total Score [20]
Ling et al. [1]	1	4	0	1	1	2	1	1	1	1	1	1	1	1	17
Abalo- Núñez et al. [2]	1	4	0	0	1	2	0	0	1	1	0	1	1	1	13
Linder and Caine [4]	1	4	0	1	1	1	1	0	1	1	0	1	1	1	14
Sweeney et al. [9]	0	4	1	1	1	1	0	0	1	1	0	1	1	1	13
Bukva et al. [21]	1	4	0	1	1	2	1	0	1	0	0	1	1	1	14
Miller et al. [22]	0	4	1	1	1	2	1	0	1	0	1	1	1	1	15
Cupisti et al. [23]	0	4	0	1	1	2	0	0	1	0	0	1	1	0	11
Toraman et al. [24]	0	4	0	1	1	2	1	0	1	0	0	1	1	1	13
DiFiori et al. [25]	0	4	1	1	1	2	0	0	1	1	0	1	1	1	14
Kirkby et al. [26]	0	4	0	0	1	2	0	0	1	0	0	0	1	1	10
Steele and White [27]	0	4	0	0	1	2	0	0	1	1	0	0	1	1	11
Vanti et al. [28]	0	4	1	1	1	0	0	0	1	1	0	0	1	1	11
Wright and De Crée [29]	0	4	0	1	1	1	1	0	1	0	0	1	1	1	12
Ghasempour et al. [30]	0	4	1	1	1	2	1	1	1	0	1	0	1	0	14
Ghasempour et al. [31]	0	4	1	1	1	2	1	1	1	0	1	0	1	0	14

The maximum possible score for quality was 20, this score was derived from 14 domains

^a^Study design (1pt = prospective, 0pt = retrospective)

^b^Level of evidence (Oxford Centre for Evidence Based Medicine Levels of Evidence: level 1 = 5 pts; level 2 = 4 pts; level 3 = 3 pts; level 4 = 2 pts; level 5 = 1 pt), ^c^Selection criteria (inclusion and exclusion criteria were clearly described = 1 pt)

^d^Setting (enough information was provided to identify the setting = 1pt)

^e^Demographic information (age (mean or median and SD or range) and gender were reported = 1pt)

^f^Description of the screening tool (test device or instruments = 1pt, protocol of screening tool(s) reported = 1pt, insufficient data to permit replication of the test)

^g^Injury definition (clear and appropriate definition is provided = 1pt)

^h^Injury diagnosis (made by physical therapist/ physiotherapist or doctor = 1pt, self-assessed = 0pt)

^i^Statistical analysis (detail given on mean or median, SD, P value or CI = 1pt)

^j^Predictive statistical analysis (multivariate regression analysis or RR/OR used as predictive value = 1pt)

^k^Reliability of index test (reliability reported from previous research = 1pt, reliability reported from actual study data = 2pts)

^l^Percentage missing (all included subjects measured and if appropriate missing data or withdrawals from study reported or explained = 1 pt)

^m^Outcome (outcome clearly defined and method of examination of outcome adequate = 1 pt)

^n^Confounder (most important confounders and prognostic factors identified and adequately taken into account in design study = 1 pt)

### Data Analysis

A meta-analysis was considered for all outcome measures. Four studies reported body weight for injured and non-injured gymnasts [4, 9, 23, 25]. However, the age of gymnasts in these studies ranged from 5 to 19 years and hence it was not appropriate to pool data. Age differences in the samples were also the reason for not pooling height data provided by 3 studies [4, 9, 25] and waist circumference data from two studies [4, 23]. Grip strength of gymnasts was measured by [4, 25]; however, different data analysis procedures were used, only one study normalised the scores by body weight [4], and hence again, pooling was not possible. Finally, vertical jump [1, 4, 29] and quadriceps angle (Q angle) data [2, 24] were considered for meta-analysis; however, inconsistency in protocols and data presentation prevented pooling of the data.

## Results

### Included Studies

**Fig. 1:**
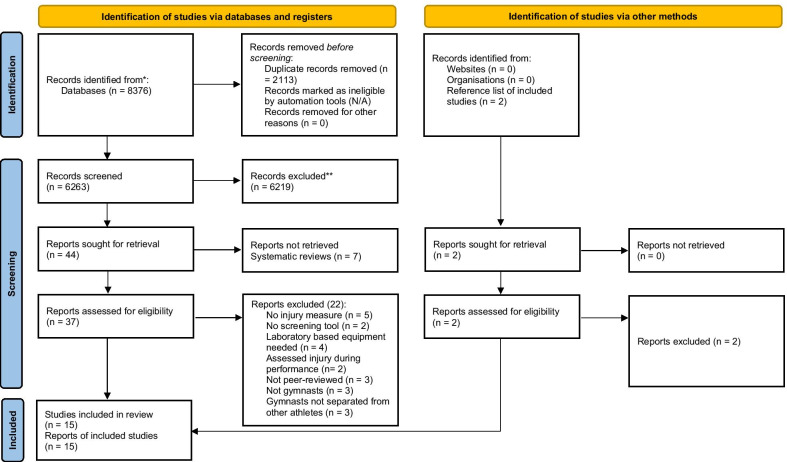
Prisma diagram of search strategy

The initial search yielded 8376 studies for review. The title and abstracts of these articles were reviewed, and duplicates removed, which resulted in 6263 articles requiring further consideration. Assessment of the eligibility of the full text of these articles and the application of inclusion and exclusion criteria meant that 15 articles were included in the systematic review. Figure 1 outlines the search strategy [32]. The assessment of the methodological quality is reported in Table 1. The mean score was 13.1 points (range 10–17 points). Table 2 reports the characteristics of these studies.

**Table 2:** Characteristics of the studies included in the literature review

Article	Population	Screening Tools	Definition of Injury	Diagnosis of Injury	Findings
Ling et al. [1]	N = 100 female gymnasts Seven National College Athletic Association Division 1 women’s gymnastic programs in the Midwestern United States Female 19.6 ± 1.2yrs, BMI 23.1 ± 2.0	Gymnastics Functional Measurement Tool and 10 associated items Rope climb Vertical jump Hanging pikes Shoulder flexibility angle Agility sprint time Over-grip pull-ups Split sum Push-ups 20 yard sprint time Handstand hold time	Gymnastics-related injury “*an injury that resulted from practice, competition, or conditioning that caused the athlete to modify or stop participation and required attention from a medical provider (certified athletic trainer, physical therapist, physician or chiropractor*” Previous injuries were those that occurred before participants were enrolled in the study Current injury was an injury that occurred during a single academic year Injury: “*Any gymnastics related physical damage causing the gymnast to miss or modify one or more training sessions, competitions or both*” Injury severity Mild: Missed ≤ 10 practices Moderate: Missed 10–25 practices Severe: Missed more than 25 practices and more than one competition and/or required surgery Injuries were categorised as: Mild: Missing 1–9 practices but no competitions Moderate: Missing 10 to 25 practices or one competition Severe: Missing > 1 competition	Assessment by medical provider certified athletic trainer, physical therapist, physician or chiropractor	Seventy-eight gymnasts sustained an injury (78%) Most injuries were in the upper extremity The most common type of injury included sprains and strains For trunk injuries, a higher score on the vertical jump test was significantly associated with a decrease in trunk injuries (OR 0.69, 95% CI 0.52–0.91, *P* = 0.01) This significant finding remained after adjusting for age, BMI, years of competition, previous injury, previous surgery and previous fracture (OR 0.69, 95% CI 0.47–1.01, *P* = 0.05) Each one point increase in vertical jump score corresponding to an additional 3.4 cm in jump height reduced the risk of trunk injury by 30% No other screening measures were associated with injury
Abalo- Núñez et al. [2]	N = 73 athletes N = 51 experimental group (gymnasts, 45 female, 6 male) N = 22 control group (athletes from other sports, all female) 56.86% gymnasts competed in national competitions 39.22% competed in international competitions 3.92% competed in regional competitions 45.45% of the control group competed at national level 4.55% of the control group competed at international level Gymnasts 13.61 ± 4.59yrs, 1.50 ± 0.15 m, 45.28 ± 14.42 kg Athletes 14.59 ± 3.93 yrs, 1.46 ± 0.10 m, 49.73 ± 11.19 kg	Q angle Perimeter of the thigh	Injury at the end of the season (injury/no injury) No definition of injury provided	Injury questionnaire	Twelve gymnasts reported injury in the season Significant differences between injured and uninjured gymnasts for mean right Q angle (*P* = 0.005), mean left Q angle (*P* = 0.003), with a greater Q angle potentially predisposing to injury Significant differences between injured and uninjured gymnasts for mean right bilateral weight-bearing on right leg (*P* = 0.025) Age (*P* = 0.012), weight (*P* = 0.028) and height were significant variables for injury incidence rate. The effect of the Q angle on the probability of injury varies depending on the gymnast’s weight (*P* = 0.026) An excessive Q angle may predispose to injury
Linder and Caine [4]	N = 68 females Competitive gymnasts from 3 prominent private clubs (beginner to international level) Comparison of 27 injured and 41 non-injured gymnasts Low level: Canadian provincial level 1,2 and 3 High level: Canadian provincial level 4 and national elite level Injured (whole sample) 12.3 ± 1.88 yrs, 145.29 ± 11.00 cm, 37.87 ± 8.16 kg Non-injured (whole sample) 10.91 ± 1.60 yrs, 137.93 ± 10.01 cm, 32.43 ± 6.49 kg Low-level injured 11.24 ± 1.57 yrs, 143.05 ± 12.13 cm, 35.80 ± 8.40 kg Low-level non-injured 10.49 ± 1.51 yrs, 136.31 ± 10.31 cm, 31.33 ± 6.15 kg High-level injured 13.09 ± 1.75 yrs, 147.69 ± 7.67 cm, 40.11 ± 7.59 kg High-level non-injured 11.93 ± 1.35 yrs, 140.98 ± 10.24 cm, 35.10 ± 6.75 kg	Height Weight Biepicondylar femur width Thigh circumference Wrist circumference Total skinfold (sum of 6 skinfolds) Endomorphy Mesomorphy Ectomorphy Shoulder flexion strength Grip strength right Pronation/supination flexibility Splits forward right Sit and reach Leg raise forward left 20 m run Leg lifts Standing broad jump Bar dips Vertical jumps	Injury rate: the number of injuries sustained during the surveillance period/estimated number of hours trained during that period × 100 Time lost: number of practice hours affected by injury estimated from the injury follow-up report and the gymnast’s competitive level/ total number of hours trained: Served as a measure of seriousness of the injury The definition of injury referred to a previous publication; however, the actual definition was not given within the article	Method of collecting injury information and definition of injury described previously in another publication This article does not state who collected data or how	Twenty-seven gymnasts reported an injury 14 low level and 13 high level Injured gymnasts were significantly taller (*P* < 0.01) and heavier (*P* < 0.01) than non-injured in whole sample Biepicondylar width higher for injured gymnasts (*P *< 0.01) in whole sample Thigh circumference higher for injured gymnasts (*P* < 0.001) in whole sample Wrist circumference higher for injured gymnasts (*P* < 0.02) in whole sample Shoulder flexion strength higher for injured gymnasts (*P* < 0.05) in whole sample Shoulder flexion strength higher for injured gymnasts (*P* < 0.05) in low level Grip strength right higher for injured gymnasts (*P* < 0.01) in whole sample Grip strength right higher for injured gymnasts (*P* < 0.05) in high level Injured gymnasts had significantly greater splits forward right (*P* < 0.05) in low level Injured gymnasts had significantly lower splits forward right (*P* < 0.02) in high level Injured gymnasts had significantly better sit and reach (*P* < 0.05) in the whole sample Injured gymnasts had significantly better sit and reach (*P* < 0.05) in the high-level sample Injured gymnasts had significantly higher leg raise forward left (*P* < 0.02) in the whole sample Injured gymnasts had significantly better leg raise forward left (*P* < 0.05) in the low level Injured gymnasts had significantly greater 20 m run time (*P* < 0.01) in the whole sample Injured gymnasts had significantly greater bar dips (*P* < 0.05) in the high level Injured gymnasts had significantly greater vertical jump (*P* < 0.01) in the whole sample Body size, strength, power and speed identified as significant discriminating variables in whole sample and twice less reliably in the competitive levels In all three instances, the injured gymnasts had higher class means than non-injured Higher injury rate for high-level gymnasts scoring low on balance, speed and arm flexibility/strength
Sweeney et al. [9]	N = 67 female gymnasts 6 to 18 years old Level 3 to 10 of the USA Gymnastics Junior Olympic Programme Participants with low back pain (N = 30): 13.7 ± 2.8 yrs, 149.1 ± 14.5 cm, 43.4 ± 12.3 kg Participants without low back pain (N = 37): 11.7 ± 2.8 yrs, 143.2 ± 13.9 cm, 37.5 ± 11.3 kg	Active and passive shoulder flexion Active and passive hamstring flexibility (popliteal angle) Active and passive prone hip extension Active and passive prone knee flexion (quadriceps flexibility) Thomas test	No definition of injury provided Gymnasts to report their history of low back pain within the past 12 months If yes then the months were determined and whether they had seen a medical provider	Self-reported via questionnaire	Thirty gymnasts reported lower back pain (44.8%) Gymnasts with lower back pain were older and heavier Negative left Thomas Test was independently associated with low back pain (*P* = 0.03) and therefore gymnasts with a positive test were less likely to report lower back pain Right passive prone hip extension was lower in the back pain group (*P* = 0.04) No other flexibility measurement independently associated with increased risk of low back pain
Bukva et al. [21]	N = 24 (7 males) Qatar National Team artistic gymnasts Aged 11–26 yrs Males 16.29 ± 4.88 yrs Females 13.86 ± 2.85 yrs	Beighton score	Time lost from participation (practice and competition) during 2015/16 season	*“Certified trainers were recruited to prospectively record injury data”*	No correlation between hyperelasticity score and number of injuries No correlation between training hours per week and number of injuries
Miller et al. [22]	N = 55 (28 females) British Gymnastics Lilleshall National Sports Centre during national squad training camp Member of National Gymnastics Artistic Squad Males 19.2 ± 3.5 yrs Females 16.9 ± 3.0 yrs Injured 18.7 ± 3.2yrs Uninjured 17.0 ± 2.8yrs	Weight-bearing dorsiflexion was measured using a weight-bearing lunge test	Defined as injured (previous ankle injury) and non-injured (no previous ankle injury) No current ankle injury	Self-reported via questionnaire	There were 48 injured limbs Injured limbs had a smaller range of weight-bearing dorsiflexion with greater variability compared to uninjured (44.8° ± 6.1°, 95% CI 43.0°, 46.5°for injured and 45.4° ± 6.1, 95% CI 43.9°, 46.9°for uninjured) Injured limbs had a smaller range of weight-bearing dorsiflexion compared to the uninjured limbs, with the injured limbs having greater variability (uninjured 47.4° ± 5.7°, 95% CI 45.0°, 49.8°; injured 45.1° ± 6.0°, 95% CI 42.6°, 47.7°; *t*(23) = −3.259, *P* = 0.03)
Cupisti et al. [23]	N = 171 females 67 rhythmic gymnasts 104 controls 19 clubs, affiliated members of the Italy Federation of Gymnastics Rhythmic gymnasts no low back pain complaint 14.5 ± 2.1 yrs, 46.8 ± 7.6 kg, BMI 18.4 ± 1.9 Rhythmic gymnasts low back pain 15.4 ± 1.5 yrs, 51.8 ± 3.7 kg, BMI 19.5 ± 0.9 Controls no complaint 14.5 ± 1.6 yrs, 53.2 ± 9.9 kg, BMI 20.5 ± 3.1 Controls low back pain 15.4 ± 1.8 yrs, 59.7 ± 9.7 kg, BMI 22.6 ± 3.6	Four skinfold thickness measurements (triceps, biceps, inter-scapular and suprailiac taken using Holtain’s skinfold calipers to calculate body fat density Waist circumference	“*Yes-response to the question do you often have back pain? was defined as having back pain"*	Self-reported by questionnaire with intensity, location and characteristic of pain defined using categorical and continuous (0 to 10 rating) items	Seven out of 67 gymnasts reported low back pain (10.4%) Six gymnasts reported bilateral pain and one central low back pain In both gymnasts and controls the symptom-free females demonstrated lower body weight (*P* < 0.05), lower BMI (*P* < 0.05), lower fat body mass (*P* < 0.05) and lower waist circumference (*P* < 0.01) than those complaining of low back pain
Toraman et al. [24]	N = 32 females 17 girls attending the School of Physical Education and Sports and participating in a rhythmic gymnasts course 15 girls (controls) attending the Tourism Institution of Higher Education Ballet students attending intensive summer ballet programmes Gymnasts 20.4 ± 1.5 yrs, 166.6 ± 5.2 cm, 55.2 ± 4.7 kg Controls 19.7 ± 0.8 yrs, 167.3 ± 3.2 cm, 54.5 ± 3.6 kg	New York Posture Rating Test Screens posteriorly (Scored/30): Head Vertebral column Pelvis Heels and foot bases Screens laterally (scored/35) Neck Breast Shoulders Back Body Abdominal protrusion and waist Other measurements: Genu valgum Genu Varum Tibial torsion Q angle Feiss line	Acute injuries *“sudden appearing, severe injuries”* Chronic injuries *“result of repetitive low threshold forces, which decreased with activity and warm-up and where pain increased following activity”*	Self-reported retrospectively via questionnaire “*Measurements and evaluations were made by two different investigators*”	Four gymnasts had acute and ten chronic injuries of ankle and / or foot Unclear regarding other injuries Gymnasts having normal foot and ankle appearance had no injury history (*P* < 0.05) 79% of subjects with pes planus had an ankle injury history Gymnasts had no knee injuries so variations in Q angle were not investigated
DiFiori et al. [25]	N = 59 (31 males) Single gymnastic club Precompetitive level (N = 26) Beginning level (N = 26) Middle level (N = 5) Advanced level (N = 2) Elite level (N = 0) Males 9.3 ± 2.3 yrs, 129.3 ± 13.8 cm, 29.0 ± 7.4 kg Females 9.4 ± 2.5 yrs,129.6 ± 16.2 cm, 28.8 ± 10.0 kg	Bilateral grip strength	Quality, location and duration of wrist pain in the previous 6 months Training sessions missed and number of days per month missed recorded	Questionnaire used to record wrist pain	Thirty-three gymnasts reported wrist pain (56%) 22 gymnasts had bilateral pain (67%) Thirty-six per cent of those with wrist pain (12 of 33) had symptoms that interfered with training No significant difference in grip strength for both left and right wrist between those with and without wrist pain in absolute grip strength and when adjusted for weight for both males and females
Kirby et al. [26]	N = 95 (60 female gymnasts, 35 female controls) Competitive-level gymnasts Age matched nonathletic girls Gymnasts 11.8 ± 2.5 yrs 144.8 ± 13.0 cm 36.6 ± 9.8 kg Controls 11.7 ± 2.1 yrs 148.8 ± 11.7 cm 42.2 ± 9.4 kg	Shoulder flexion Shoulder horizontal abduction Elbow extension Elbow supination Lumbar motion Hip extension Knee extension Toe touching	Participants asked to describe current or past musculoskeletal symptoms including *“broken bones, spasms, swelling, snaps”* Enquired regarding specific regions Categorised severity as: Mild: Symptoms only Moderate: Symptoms and signs Severe: Required physician visit Categorised duration as: Short: Less than 1 week Medium: 1 week to 1 month Long: Greater than 1 month	Reported via interview When symptoms identified one of the investigators performed a musculoskeletal examination	Total number of injured gymnasts is not reported No significant differences between gymnasts and controls in the severity or duration of symptoms Average number of symptomatic areas per participant was 2.25 for controls and 6.17 for gymnasts (*P* < 0.001) Gymnasts who had greater ROM on toe touching also had greater low back pain (*P* = 0.043)
Steele and White [27]	N = 40 females Competitive gymnasts Local gymnastic clubs and the zone squad of the North of England 10–21 yrs Low injury status N = 10 10.8 ± 0.33 yrs(SE) 135.2 ± 1.62 cm(SE) 31.8 ± 1.14 kg(SE) High injury status N = 10 14.6 ± 0.83 yrs(SE) 153.4 ± 2.65 cm(SE) 46.9 ± 3.02 kg(SE)	Hypermobility assessed using Carter and Wilkinson method modified by Beighton and Horan Peripheral flexibility determined using a Leighton flexometer including: Shoulder flexion with elevation, shoulder abduction with elevation, elbow and wrist flexion, hip flexion with knee extension, flexion with knee flexion and abduction, knee flexion and ankle dorsiflexion with knee flexion/extension Total peripheral flexibility score was obtained by the sum of all peripheral joint flexibility scores Thoracic and lumbar curvatures in standing and lumbar extension in prone were measured using a Loebl hydrogoniometer Height Weight Endomorphy, mesomorphy and ectomorphy determined using modified somatotype method of Heath and Carter	Injury score categorised injuries in terms of severity whereby numerical ratings were assigned from 1 to 20 No apparent injury definition	Unclear how injury was diagnosed	Significant differences between the low injury and high injury group were reported for age (*P* < 0.001), weight (*P* < 0.001), Quetelet index (*P* < 0.001), lumbar extension (*P* < 0.05) and shoulder flexion (*P* < 0.005) Significant positive relationship between injury score and weight (*P* < 0.05), lumbar curve (*P* < 0.05), age (*P* < 0.001) Significant negative relationship between injury score and mesomorphy (*P* < 0.05) and height (*P* < 0.05) Weight, mesomorphy, standing lumbar curvature, age and height (*R* = 0.834, *R*^2^ = 0.696) were significant predictors of injury score
Vanti et al. [28]	Main gymnastic clubs of Emilia-Romagna Region, Italy Adolescents attended junior high school in 3 institutes of the Emilia-Romagna Region N = 466 (91 club-level gymnasts, 85 females, 6 males) 12.3 ± 3.63, 144.9 ± 12.2 cm, 38.5 ± 12.1 kg, BMI 17.9 ± 2.62 375 adolescents (173 females, 202 males) 13.07 ± 0.95 yrs, 154.7 ± 16.3 cm, 49.8 ± 11.7 kg, BMI 20.3 ± 3.4	Height Weight BMI Lumbar ROM (via electronic motion evaluation system) therefore not included in review analysis	No definition of injury provided Study assessed pain via a questionnaire *Have you ever had back pain and with what frequency?* *How would you rate your usual pain from 0 to 10?* Divided into low-level low back pain frequency of “*sometimes*” Medium/high-level “intensity ≥ 4/10”	Self-reported via questionnaire	Forty-two (46%) and 24 (26%) gymnasts reported low-level and high-level lower back pain respectively No significant difference between height, weight, BMI and LBP in either the gymnasts or adolescent groups
Wright and De Crée [29]	N = 15 females Members of the Kettering Olympic Gymnastic Club Elite Competition Squad, UK Age range 8 to 18 years old 11.8 ± 3.5 yrs Low injury 9.5 ± 1.3 yrs, Height 132 ± 6.8 cm, mass 28.0 ± 4.3 kg, BMI 15.8 ± 1.3 High injury 14.3 ± 3.3 yrs, Height 153.9 ± 17.3 cm, Mass 48.0 ± 12.8 kg, BMI 15.0 ± 0.5	Somatotype determined using the Heath-Carter somatotype method (categorised as endomorph, mesomorph or ectomorph) Percentage body fat estimated from 4 subcutaneous skinfolds (triceps, subscapular, suprailiac, medial calf) Height Mass Biceps muscle girth Medial calf muscle girth Humerus bone width Femur bone width Grip strength Standing vertical jump Muscle endurance via pull-ups and push-ups Sit and reach Shoulder and wrist elevation Back extension in bridge Ankle dorsiflexion Ankle plantarflexion	*“A gymnastics-related incident that limited participation in any of the gymnastic events”* Participants classified as having a *“low”* or *“high”* injury status based upon previously described system in [27]	Self-reported by questionnaire for injuries in the previous 4 years	Low injury *N* = 8, high injury *N* = 7 Abrasions were most common in the low injury group and sprains in the high injury group The high injury group was significantly older (*P* = 0.002), taller (*P* = 0.006), heavier (*P* = 0.001) Significant difference in BMI (*P* = 0.001) with the high injury group being marginally *“underweight”* and the low injury group had *“severe protein-energy malnutrition”* High injury group scored better on the vertical jump than the low injury group (*P* = 0.021) Low injury group scored better on back extension in the bridge (*P* = 0.013) and ankle dorsiflexion (*P* = 0.013) The number of overuse injuries was significantly higher in the high injury group than in the low injury group (29.0% vs. 11.1%)
Ghasempour et al. [30]	N = 43 Elite male gymnasts in the Iranian Premier League and Division One Age 16 to 28 years (mean 20.47 yrs) Weight 64.33 ± 7.2 kg Height 170 ± 0.05 cm BMI 22.15 ± 2	Weight Height BMI Endomorphy Mesomorphy Ectomorphy Fat percentage Ankle girth Calf girth Length of lower extremity Small body size Medium body size Large body size Total body size	Injury was defined as *“any damaged body part (only the ankle in this study) that required medical attention or prevented or restricted the gymnasts from training or competing in any activity/apparatus in any way and/or length of time”*	Gymnasts completed an injury questionnaire while a sports specialist was available to answer any questions	79% of gymnasts had experienced ankle injuries over the past year Joint and ligamentous injuries were the most common Body size had a positive relationship with ankle injuries (*P* = 0.002, *r* = 0.524) There was no significant relationships between the presence of ankle injuries and other anthropometric characteristics
Ghasempour et al. [31]	N = 43 Elite male gymnasts in the Iranian Premier League and Division One Weight 64.33 ± 7.2 kg Height 170 ± 0.05 cm BMI 22.15 ± 2 Age 16 to 28 years (mean 20.47 yrs)	Weight Height BMI Endomorphy Mesomorphy Ectomorphy Fat percentage Upper extremity length Wrist girth Forearm girth Small body size Medium body size Large body size Total body size	Injury was defined as* “any damaged body part (only the wrist in this study) that required medical attention or prevented or restricted the gymnasts from training or competing in any activity/apparatus in any way and/or length of time”*	Gymnasts completed an injury questionnaire while a sports specialist was available to answer any questions	53.5% of gymnasts had experienced a wrist injury over the last year Skin and muscular injuries were the most common Weight was positively related to wrist injuries (*P* = 0.02, *r* = 0.34)

### Gymnastic Genre and Level

Two studies included artistic gymnasts [21, 22], 2 studies included rhythmic gymnasts [23, 24], 11 studies simply used the term “gymnasts” [1, 2, 4, 9, 25–31]. With regard to the level of gymnasts, 6 studies included gymnasts classified as national team level [9, 21, 22, 29–31], 2 studies involved gymnasts from clubs affiliated with a national federation [1, 23], 1 study involved gymnasts from a precompetitive level [25], 3 studies used competitive-level gymnasts [4, 26, 28], 2 studies used a mixed group of gymnasts [2, 27] and 1 study included gymnasts attending a course and the level was unclear [24].

### Age and Sex

Four studies used gymnasts under 18 years old [4, 25, 26, 28] and 2 studies included gymnasts above 18 years old [1, 24]. Four studies included gymnasts across the age range; 6–18 years [2, 9, 21, 22], 1 study 13–19 years [23], 1 study 10–21 years [27], 1 study 8–18 years [29] and 2 studies 16–28 years [30, 31]. Seven studies included females only [1, 4, 9, 23, 24, 26, 27], 6 studies were mixed [2, 21, 22, 25, 28, 29] and 2 studies included males only [30, 31].

### Injury Definition and Diagnosis

Eight studies provided a definition of injury [1, 4, 21, 22, 24, 29–31] and 7 studies did not define injury [2, 9, 23, 25–28]. Four studies investigated/defined pain [9, 23, 25, 28]. In 5 studies [22, 24, 29–31] injury was self-reported, and in 4 studies pain was self-reported [9, 23, 25, 28]. In 3 studies [2, 4, 27] the method of diagnosis was unclear. In 1 study certified trainers recorded injury data [21] and in 1 study the “researchers” assessed the injury [26]. In one study, the diagnosis was provided by a “certified athletic trainer, physical therapist, physician or chiropractor” [1]. Seven studies investigated a specific type of injury/pain: low back pain [9, 23, 28], wrist pain [25], ankle injury [22, 30] and wrist injury [31].

### Statistical Analysis

Seven studies used regression models or risk measurement [1, 2, 4, 9, 25, 27, 28], and 8 studies used inferential analysis that did not include regression or risk measurements [21–24, 26, 29–31]. Six studies used both types of statistical analysis [2, 4, 9, 25, 27, 28].

### Range of Motion

Eight studies [4, 9, 22, 24, 26–29] investigated the relationship between range of motion (ROM) and injury. Only the screening data for significant results are reported here.

Kirby et al. [26] investigated 60 female gymnasts and 35 aged matched non-athletic controls for musculoskeletal symptoms and flexibility ROM and reported that those gymnasts who had greater ROM on toe touching also had greater low back pain (*P* = 0.043) when compared with the controls. Lindner and Caine [4] reported that injured gymnasts had better sit and reach (*P* < 0.05) and higher leg raise forward left (*P* < 0.02) results than uninjured gymnasts but were not different in the remaining flexibility tests. When gymnasts were separated into high- and low-level performance groups, injured gymnasts in both groups had significantly greater forward splits on the right side (where the right leg is extended forward and the left leg positioned to the rear of the trunk) compared with uninjured gymnasts (high level *P* < 0.02, low level *P* < 0.05). In the low-level group only, injured gymnasts had significantly higher leg raise forward on the left side (*P* < 0.05). Injured high-level gymnasts had better sit and reach scores than high-level uninjured gymnasts (*P* < 0.05). Finally, high-level injured gymnasts who scored lower on a combination of balance, speed and arm flexibility/strength variables had higher injury rates (*P* = 0.0495) in the stepwise regression model.

Miller et al. [22] reported that injured limbs had a smaller range of weight-bearing ankle dorsiflexion with greater variability compared to uninjured (44.8° ± 6.1°, 95% confidence interval (CI) 43.0°–46.5° for injured and 45.4° ± 6.1°, 95% CI 43.9°–46.9° for uninjured) in 55 national-level artistic gymnasts. Wright and De Crée [29] reported that in 15 gymnasts the lower injury group scored lower on ankle dorsiflexion (62.3 cm ± 8.0 vs. 75.9 cm ± 10.3, *P* = 0.013) and back extension in the bridge (22.6 cm ± 1.9 vs. 25.5 cm ± 4.9, *P* = 0.013) which they stated as better performances when compared with the high injury group. Steele and White [27] reported low injury groups had superior scores for lumbar extension; 46.7° ± 2.94 (mean ± standard error (SE)) versus 59.8° ± 4.32 (mean ± SE) (*P* < 0.05) and shoulder flexion 253.8° ± 3.13(SE) versus 240.1° ± 4.91(SE) (*P* < 0.05) when compared to high injury groups, respectively, in 40 North of England squad female gymnasts.

### Anthropometrics and Posture

Nine studies investigated the relationship between anthropometric values and/or posture and injury [2, 4, 23, 24, 27–31]. Only the screening data for the significant results are reported here.

In a study investigating low back pain in 67 female rhythmic gymnasts and 104 controls, skinfold measurements were obtained at the triceps, biceps, inter-scapular and suprailiac to calculate body density [23]. In both gymnasts and controls the symptom-free females demonstrated lower body weight (*P* < 0.05), lower body mass index (BMI), *P* < 0.05), lower fat body mass (*P* < 0.05) and lower waist circumference (*P* < 0.01) than those with lower back pain [23]. Lindner and Caine [4] reported that in 68 competitive gymnasts, injured gymnasts were significantly taller (*P* < 0.01) and heavier (*P* < 0.01) than non-injured. Injured gymnasts had significantly greater biepicondylar femur width (*P* < 0.01), thigh circumference (*P* < 0.001), wrist circumference (*P* < 0.02) in the whole sample (*P* < 0.01) and a significantly greater biepicondylar femur (*P* < 0.05) and thigh circumference (*P* < 0.01) in high-level gymnasts. Thigh circumference (*P* < 0.01) and wrist circumference (*P* < 0.02) were significantly greater in injured gymnasts in the low-level sample.

Abalo-Núñez et al. [2] reported that in 51 national- and international-level gymnasts, age (*P* < 0.01), weight (*P* < 0.01) and height (*P* < 0.01) were significant variables for injury incidence rate. In 40 female competitive gymnasts, height, weight and somatotype were determined and significant differences existed between low injury status and high injury status groups for age (*P* < 0.001), weight (*P* < 0.001) and BMI (*P* < 0.001) [27]. There was a significant positive relationship between injury score and weight (*P* < 0.05) and age (*P* < 0.001) and a significant negative relationship between injury score and mesomorphy (*P* < 0.05) and height (*P* < 0.05). Postural measurements of thoracic and lumbar curvatures and lumbar extension were taken using a Loebl hydrogoniometer and a significant difference existed between the low injury and high injury group for lumbar extension (*P* < 0.05), and there was a significant positive relationship between injury score and lumbar curve (*P* < 0.05). Unfortunately, it was unclear how body size was measured in this paper. Abalo-Núñez et al. [2] also reported a significant difference between injured and uninjured gymnasts for mean right *Q* angle (*P* = 0.005), mean left *Q* angle (*P* = 0.003) and the effect of *Q* angle on the probability of injury varied depending upon the gymnast’s weight (*P* = 0.026).

Toraman et al. [24] investigated posture using the New York Posture Rating Test which screens various parts of the body in 17 female gymnasts and a sedentary female control group attending a rhythmic gymnastics course and reported that gymnasts with normal foot and ankle appearance had no injury history (*P* < 0.05) while 79% of “subjects” with pes planus had an ankle injury history. However, the number of gymnasts within this percentage was not reported. Wright and DeCrée [29] determined somatotype and BMI utilising a 4-skinfold method at the triceps, subscapular, suprailiac and medial calf in 15 international-level gymnasts and classified injury status as low or high. The high injury group was significantly older (*P* = 0.002), taller (*P* = 0.006), heavier (*P* = 0.001) and there was a significant difference in BMI (*P* = 0.001) with the high injury group marginally “underweight” and the low injury group demonstrating “severe protein energy malnutrition”; however, the authors did not define these terms.

Ghasempour et al. [30] investigated ankle injuries in 60 elite male gymnasts of which injury data were recorded for 40 gymnasts. They reported that body size had a significant positive relationship with ankle injuries (*P* = 0.02, *r* = 0.524). Ghasempour et al. [31] investigated wrist injuries in 43 elite male gymnasts and reported that body weight had a significant positive relationship (*P* = 0.02, *r* = 0.34) and that heavier gymnasts suffered more injuries.

### Hypermobility

Two studies reported no significant relationship between hypermobility scores, measured using the Beighton modification of the Carter–Wilkinson criteria (cut-off ≥ 5), and injury [21, 27].

### Clinical Diagnostic Tests

One study [9] investigated the relationship between clinical diagnostic tests and injury but only used one clinical diagnostic test, namely the Thomas test alongside a series of ROM measurements. Only the screening data for significant results are reported here.

A negative left Thomas test was independently associated with low back pain (*P* = 0.03), and therefore, gymnasts with a positive test were less likely to report low back pain.

### Movement Screening Tools

One study investigated the relationship between movement screening tools and injury [1]. This study used the Gymnastics Functional Measurement Tool which consists of 10 items and investigated 100 female gymnasts. Only the screening data for significant results are reported here.

For trunk injuries a higher score on the vertical jump test was significantly associated with a decrease in trunk injuries (OR 0.69, 95% CI 0.52–0.91, *P* = 0.01) and for each one point increase in vertical jump score which corresponded to an additional 3.4 cm the risk of trunk injury was reduced by 30%.

### Muscle Control, Strength, Power and Endurance

Three studies investigated muscle control, strength, power and endurance [4, 25, 29]. Only the screening data for significant results are reported here.

DiFiori [25] investigated the relationship between bilateral grip strength and wrist pain in 59 precompetitive-level gymnasts and reported no significant difference between those with and without wrist pain in relation to absolute grip strength and when adjusted for weight for both males and females. Lindner and Caine [4] compared 27 injured and 41 non-injured gymnasts and reported shoulder flexion strength was greater in injured gymnasts for the whole sample (*P* < 0.05) and low-level sample (*P* < 0.05). Grip strength right for the whole sample (*P* < 0.01) and high-level sample (*P* < 0.05) was greater in injured gymnasts compared to uninjured gymnasts. Injured gymnasts had significantly greater bar dips in the high-level gymnasts (*P* < 0.05) and significantly greater vertical jump (*P* < 0.001) in the whole sample. In addition, strength, power and speed were identified as significant discriminating variables with injured gymnasts having higher class means than non-injured. Wright and De Crée [29] reported that the “high injury status” group had a better vertical jump than the “low injury status” group (*P* = 0.021).

### Other Screening Measurements

One study investigated 20 m run time [4] and injured gymnasts had significantly faster times in the whole sample (*P* < 0.01) compared with uninjured gymnasts. All other findings were reported at a non-significant level.

## Discussion

To the best of our knowledge, this is the first systematic literature review to investigate which screening tools can predict injury in all genres, levels and ages of gymnasts.

### Methodological Quality

The mean score using the methodological quality tool was 13.1 points (range 10–17 points) with all studies being of level 4 evidence. All studies provided a description of the screening tools used. As with many injury studies, the literature is limited by the varying definitions of musculoskeletal injury and by who defined the injury. Eight studies provided a definition of injury [1, 4, 21, 22, 24, 29–31] and 7 studies did not define injury [2, 9, 23, 25–28]. Seven studies investigated a specific type of injury/pain: low back pain [9, 23, 28], wrist pain [25], ankle injury [22, 30], wrist injury [31]. Four studies investigated/defined pain [9, 23, 25, 28]. In 5 studies [22, 24, 29–31] injury was self-reported, and in 4 studies pain was self-reported [9, 23, 25, 28]. In 3 studies [2, 4, 27] the method of diagnosis was unclear. In 1 study certified trainers recorded injury data [21] and in 1 study the “researchers” assessed the injury [26]. In one study, the diagnosis was provided by a “certified athletic trainer, physical therapist, physician or chiropractor” [1]. As a minimum, it is recommended that studies should provide a definition of musculoskeletal injury and have the diagnosis made by a medical professional ideally a physical therapist/physiotherapist or doctor as self-reporting by athletes has a greater potential for misdiagnosis [18].

The reporting of the reliability of the screening tools used is important and was reported in 2 studies [1, 22] but no researchers reported the reliability within their own study. Data comparison was limited by some studies categorising gymnastic data into low-level and high-level gymnasts [4] and low injury and high injury status [27, 29]. Four studies that investigated pain [9, 23, 25, 28] were included to improve the depth of the review due to the terms often being used interchangeably; however, pain is different to injury and therefore any comparisons are required to consider the subjective nature of pain which is not always indicative or synonymous with injury. The development of chronic pain can be due to factors other than the cause of pain [33] and stress and environmental factors may require consideration. Kirby et al. [26] investigated symptoms and this requires consideration when interpreting the literature. Future research should clearly differentiate between pain and injury.

### Range of Motion

Five studies reported significant findings between ROM and injury; however, these findings were across a number of locations and comparison included different/missing musculoskeletal injury definitions, measurements taken, mix of genres, levels and ages of gymnasts.

Two studies demonstrated significant findings for ankle dorsiflexion [22, 29]. Injured limbs had a smaller range of weight-bearing dorsiflexion with greater variability compared to uninjured in artistic gymnasts [22]. However, comparison with [29] is limited as [22] only investigated ankle injuries and had a varying methodology dependent upon the objective. For objective 1, data were used for all participants and each limb was considered individually while objective 3 only used participants with a history of a unilateral previous injury. Gymnasts in the lower injury group scored better on ankle dorsiflexion and back extension in the bridge than the high injury group [29]. It was suggested that less flexible individuals might be more likely to sustain injuries.

Steele and White [27] used the same injury classification system as [29] and the finding of increased back/lumbar extension in high injury groups is in agreement. However, with these findings it is not clear if extension was measured using the same protocol. Shoulder flexion was lower in the high injury group; however, no explanation was provided for this finding [27]. The finding of Kirby et al. [26] that those gymnasts who had greater ROM on toe touching also had more low back pain may relate to shoulder flexion and back ROM as both regions contribute to this movement. However, as this was a “pain” study the complex relationship between pain and injury and different methodology restricts comparison. The sit and reach test involves back and arm ROM and [4] reported that injured gymnasts had significantly better sit and reach in the whole sample and in the high-level sample. High-level gymnasts scored low on arm flexibility and had higher injury rates. The suggestion was made that optimal levels of flexibility may exist for gymnastics with too much or too little flexibility increasing the injury risk; however, specific values were not suggested.

Due to the inconsistency of the results, it is unclear if ROM is a significant predictor of injury in gymnasts.

### Anthropometrics and Posture

Seven studies reported significant findings between anthropometric data, posture and injury [2, 4, 23, 24, 29–31]; however, these findings were across a number of locations and comparison included different/missing musculoskeletal injury definitions, measurements taken, mix of genres, levels and ages of gymnasts.

In rhythmic gymnasts and controls the symptom-free females demonstrated lower body weight, lower BMI, lower fat body mass and lower waist circumference than those with lower back pain [23]. Wright and De Crée [29] classified injury status as low or high and reported that high injury group was taller, heavier and for BMI the high injury group was marginally “underweight” and the low injury group demonstrated “severe protein energy malnutrition.” Abalo-Núñez et al. [2] reported that weight and height were significant variables for injury incidence rate and that abnormal alignments that result in unequal weight distribution may influence lower limb injury development. In female competitive gymnasts there was a significant positive relationship between injury score and weight and a significant negative relationship between injury score, mesomorphy and height [27]. It was concluded that females with poor musculature, short stature who were relatively heavy were more injury prone. Postural measurements of thoracic and lumbar curvatures and lumbar extension demonstrated a significant difference between the low injury and high injury group for lumbar extension, and there was a significant positive relationship between injury score and lumbar curve with the suggestion that hyperlordosis may predispose towards back injury [27].

Lindner and Caine [4] reported that injured gymnasts were significantly taller and heavier than non-injured in agreement with [29]. Injured gymnasts had significantly greater biepicondylar femur width, thigh circumference, wrist circumference in the whole sample and a significantly greater biepicondylar femur and thigh circumference in injured high-level compared to non-injured high-level gymnasts. Thigh circumference and wrist circumference were significantly greater in injured gymnasts than non-injured in the low-level sample. Two studies using the same participants [30, 31] highlighted that greater body size was related to ankle injuries and that greater body weight was related to wrist injuries. With ankle injuries it was suggested that increases in height and weight increase the degree of inversion torque that the ankle complex must withstand [30]. Within this study 76% of ankle injuries occurred in the landing phase and the high volume of landings associated with gymnastics increase the injury risk. For wrist injuries, heavier gymnasts are more susceptible to wrist injuries due to increased loading and because gymnastics is a sport that requires the wrist to be a wrist bearing joint [31]. However, this study did not record training or competition time or the type of apparatus used which could influence the results.

One study reported that the Q angle [2] was related to injury and it was concluded that an excessive Q angle could predispose to injury particularly the left Q angle, however, what measurement was considered an excessive Q angle was not stated. Interestingly the effect of the Q angle on injury was related to the gymnast’s weight. It was concluded that an increased Q angle may create a lateral valgus force vector that results in a misalignment of force transmission, and abnormal lateral movements and an increased risk of injury.

The interpretation of results was restricted by the different methodologies utilised which resulted in a variety of measurements been recorded. Lindner and Caine [4] and Steele and White [27] reported height, weight and body type (ectomorphy, mesomorphy, endomorphy) with no calculation of BMI. Abalo-Núñez [2] only presented height and weight with no separation of male and female data. Cupisti et al. [23] reported weight, BMI, fat body mass and waist circumference. Ghasempour et al. [30, 31] measured height, weight, BMI, fat percentage, body type (ectomorphy, mesomorphy, endomorphy) and body size and although Wright and De Crée [29] used similar variables with the exception of body size, comparison of results is limited by the age, gender and injury type differences. Therefore, based upon these methodological differences it is not possible to present specific values associated with injuries that could be used for injury prevention guidelines. However, from the limited number of studies available it is possible that taller and heavier gymnasts might be more susceptible to injury, however, what values define a tall and heavy gymnast is unclear.

### Hypermobility

Despite recognised screening tools such as the Beighton score existing, only two studies [21, 27] have investigated the relationship between hypermobility and gymnastic injury. Neither study reported any significant findings, and currently it appears hypermobility is not a significant predictor of injury in gymnastics.

### Clinical Diagnostic Tests

Only one study [9] investigated the relationship between clinical diagnostic tests (Thomas test) and reported that a negative left Thomas test was independently associated with low back pain and therefore gymnasts with a positive test were less likely to report low back pain. Potential explanations for this finding were the asymmetrical nature of gymnastics in relation to having a dominant limb potentially leading to tightness on one side, however, as leg dominance was not recorded it was acknowledged that this theory was speculative. Therefore, the evidence base is currently limited to one pain-based lower back study and requires further research.

### Movement Screening Tools

One study investigated the relationship between movement screening tools and injury [1] and reported that with the Gymnastics Functional Measurement Tool trunk injuries were reduced with a higher vertical jump score with an additional 3.4 cm reducing the risk of trunk injury by 30%. It was hypothesised that inadequate hip extensor strength might lead to a lower vertical jump height and increase vulnerability to trunk injuries; however, hip extensor strength was not measured directly. This gymnastic specific screening tool would benefit from further research to investigate the relationship with injury development further. With only one movement screening tool used this is an area for further research.

### Muscle Control, Strength, Power and Endurance

Two studies reported a relationship between muscle control, strength, power and endurance and injury [4, 29]. Lindner and Caine [4] compared injured and non-injured gymnasts and reported shoulder flexion strength (whole sample), shoulder flexion strength (low level) and grip strength right (whole sample), grip strength right (high level) were greater in injured gymnasts; however, no explanation was provided for this finding. Injured gymnasts had significantly greater bar dips in the (high level) gymnasts and significantly greater vertical jump (whole sample). It was suggested that bigger and stronger gymnasts were also older and therefore more likely to perform risky skills and practice longer although neither variable was analysed. In addition, strength, power and speed were identified as significant discriminating variables with injured gymnasts having higher class means than non-injured. This relationship between vertical jump and injury was also reported by [29] who demonstrated that the “high injury group” had a better vertical jump than the “low injury” group but is in contrast to the findings of [9].

Due to the inconsistency of results, it is unclear if muscle control, strength and power are a significant predictor of injury in gymnasts.

### Other Screening Measurements

Findings were limited to one study [4], which reported that injured gymnasts had significantly greater 20 m run speed in the whole sample; however, no discussion of this finding was provided. These findings require further investigation.

### Limitations and Recommendations for Future Research

No measurements were eligible for further analysis via a meta-analysis and the identification of which musculoskeletal screening tools may predict injury proved difficult due to the lack of standardisation of methods and data reporting. Our aim was to perform a meta-analysis of several measurements; however, this was prevented by poor clarity of methodology and variation in the measurement of parameters. Furthermore, the included literature was limited by small sample size, contrasting injury surveillance reporting and risk factor identification and failure to consider confounding variables. Some studies focussed on the identification of one specific injury type, and this should be considered when evaluating the evidence. Gymnasts often continue to compete when injured and this is particularly relevant at elite level. Gymnasts may have pain, but not necessarily be injured and this should be considered when reviewing injury and pain studies. Gymnastic performance requires sufficient energy availability to maintain health and reduce injury risk, and this is particularly important in female rhythmic gymnastics [34]. Therefore, it is recommended that future studies consider reporting nutrition intake to allow consideration of energy availability. The systematic review was limited to English language studies, and potentially, some studies may not have been included.

This study has provided information regarding the different genres, level and ages of gymnasts as all may influence study findings. Incomplete description of inclusion/exclusion criteria (9 studies) and reporting of dropouts was present (5 studies) which can restrict interpretation. Screening tool reliability requires greater consideration as an unreliable tool may result in a lack of measurement consistency and studies should consider inter and intra-rater reliability and validity. Studies should record training and competition duration to allow determination of injury rate and exposure data. Studies should report the injury severity and duration and provide an injury definition. It is also important that studies report who provides the diagnosis of injury to allow appropriate evaluation of potential clinical knowledge.

Prospective injury cohort studies are preferential in comparison with retrospective studies, and power calculations are advocated to determine sample size. However, only 4 studies were prospective. Future research should consider multivariate regression models if the aim is to determine the predictors of injury and if considering multiple risk factors should control for confounding variables and consider the potential interaction of those measures that are screened. One study [1] provided the following: (1) prospective design, (2) an injury definition, (3) a diagnosis by a physical therapist/physiotherapist or doctor and (4) the use of regression models or risk measurement. These factors represent good practice in investigating screening tools as a predictor of injury.

## Conclusions

This systematic review is the first to collate and critically appraise musculoskeletal screening tools as a predictor of gymnastic injury and uses an effective scoring tool that recognises the importance of key factors including injury reporting and reliability. Some evidence existed for measurement of height and mass within the systematic review as taller and heavier gymnasts might be more susceptible to injury. Only one study has utilised a movement screening tool, namely the Gymnastics Functional Measurement Tool. Future studies that investigate the ability of screening tools to predict injury should be prospective, use predictive statistics, report the reliability of the tests and consider confounders. A specific definition of injury should be provided and diagnosis provided by an appropriate medical professional.

## Data Availability

Data presented in this article are available in the associated studies, references are provided.

## Abbreviations

BMI: Body mass index
CI: Confidence interval
OR: Odds ratio
ROM: Range of motion
RR: Relative risk
SE: Standard error
Q angle: Quadriceps angle
